# Evaluation of Rapid Sepsityper® protocol and specific MBT-Sepsityper module (Bruker Daltonics) for the rapid diagnosis of bacteremia and fungemia by MALDI-TOF-MS

**DOI:** 10.1186/s12941-020-00403-w

**Published:** 2020-12-09

**Authors:** Léa Ponderand, Patricia Pavese, Danièle Maubon, Emmanuelle Giraudon, Thomas Girard, Caroline Landelle, Max Maurin, Yvan Caspar

**Affiliations:** 1 Laboratoire de Bactériologie-Hygiène Hospitalière, Centre Hospitalier Universitaire Grenoble Alpes, 38000 Grenoble, France; 2 Université Grenoble Alpes, CNRS, Grenoble INP, TIMC-IMAG, 38000 Grenoble, France; 3 Service de Médecine Infectieuse et Tropicale, Centre Hospitalier Universitaire Grenoble Alpes, 38000 Grenoble, France; 4 Laboratoire de Parasitologie Mycologie, Centre Hospitalier Universitaire Grenoble Alpes, 38000 Grenoble, France; 5 Service d’Hygiène Hospitalière, Centre Hospitalier Universitaire Grenoble Alpes, 38000 Grenoble, France; 6 Laboratoire de Bactériologie-Hygiène Hospitalière, Institut de Biologie et Pathologie, Centre Hospitalier Universitaire Grenoble Alpes, CS10217, 38043 Grenoble Cedex 9, France

**Keywords:** Bacteremia, Fungemia, Sepsis, Blood culture, Sepsityper®, MALDI-TOF mass spectrometry, Rapid identification

## Abstract

During bloodstream infections, rapid adaptation of empirical treatment according to the microorganism identified is essential to decrease mortality. The aim of the present study was to assess the microbiological performances of a new rapid version of the Sepsityper® kit (Bruker Daltonics) allowing identification of bacteria and yeast by MALDI-TOF mass spectrometry directly from positive blood cultures in 10 min and of the specific MBT-Sepsityper module for spectra analysis, designed to increase identification performance. Identification rates were determined prospectively on 350 bacterial and 29 fungal positive blood cultures, and compared to conventional diagnostic method. Our rapid diagnosis strategy (Rapid Sepsityper® protocol: one spot with and one without formic acid extraction step) combined to MBT-Sepsityper module provided 65.4%, 78.9% and 62% reliable identification to the species level of monomicrobial positive blood cultures growing respectively Gram-positive, Gram-negative bacteria or yeast. Importantly, identification rates of Gram-positive bacteria were higher in anaerobic than in aerobic bottles (77.8% vs 22.2%; p = 0.004), if formic acid extraction step was performed (60.8% vs 39.2%; p = 1.8e^−6^) and if specific MBT-Sepsityper module was used (76.2% vs 61.9%, p = 0.041) while no significant differences were observed for Gram-negative bacteria. For yeasts identification, formic acid extraction step improved rapid identification rate by 37.9% while the specific MBT-Sepsityper module increased overall performances by 38%, providing up to 89.7% reliable identification if associated with the standard Sepsityper® protocol. These performances, associated with a reduce turnaround time, may help to implement a rapid identification strategy of bloodstream infections in the routine workflow of microbiology laboratories.

## Background

Bloodstream infections (BSI) remain one of the major causes of death from infection in North America and Europe with a case-fatality rate between 13 and 22% [1–4]. *Escherichia coli* is the most prevalent Gram-negative (GN) pathogen followed by other species of *Enterobacterales. Staphylococcus aureus*, *Streptococcus pneumoniae* and coagulase–negative staphylococci are the most frequently isolated Gram-positive (GP) bacteria but isolation of the latter one often result from contamination by bacteria from the skin flora during the sampling process [5–7]. As regards fungi, more than 90% of BSI are caused by *Candida species*, mainly *C. albicans, C. glabrata, C. parapsilosis, C. tropicalis *and *C. krusei* [4, 8–10].

When sepsis occurs, it is essential to begin an effective and potent antibacterial or antifungal treatment as soon as possible as early administration and achievement of microbicidal concentrations are associated with better survival in community-acquired and hospital-acquired septicemia [4, 11–15]. The choice of initial large spectrum probabilistic antimicrobial therapy depends on several factors such as patient’s symptoms and medical history, recent use of antibiotics or antifungals in the previous 3 or 6 months, known carriage or suspicion of multidrug resistant (MDR) bacteria, and on local prevalence of bacterial or yeast resistance levels [16–19]. However it remains inappropriate in 20 to 40% of patients [5, 20]. Moreover, overuse of broad spectrum therapy and unnecessary treatment of contaminants may lead to adverse effects such as treatment toxicity, increased rate of post-antibiotic *C. difficile* nosocomial diarrhea episode or unnecessary hospital costs, and participates in increasing global resistance levels [21, 22]. On the opposite, antibiotic therapy de-escalation for severe sepsis and septic shock is a safe strategy associated to a reduced mortality and found as a protective factor for hospital survival [23–25].

Rapid species identification is now possible by several techniques directly from positive blood cultures. It allows a first quick adaptation of empirical treatment if inappropriate, according to the species identified [26–29]. However, available techniques are either expensive (multiplex PCR or PNA-FISH), delayed by a first 4 to 8 h subculture on agar medium or time-consuming, such as Matrix Assisted Laser Desorption Ionization—Time of Flight Mass Spectrometry (MALDI-TOF-MS) assays. In particular, despite their low cost, the important hands-on-time of MALDI-TOF-MS assays still prevents many laboratories to perform rapid identification on positive blood cultures, which results in a loss of opportunity for the patients. In-house and commercial protocols such as the Sepsityper® kit (Bruker Daltonics GmbH, Bremen, Germany) usually take between 20 to 40 min of turnaround time [22, 28, 30–43]. A comprehensive overview of current performances and estimated hands-on time of the different rapid identification methods using MALDI-TOF–MS on positive blood cultures has been gathered in Table 1.

**Table 1:** Performance of the different rapid identification methods using MALDI-TOF–MS on positive blood cultures

References	Methods			Sensibility (%)	Estimated technical time
***Commercial kits***					
[44]	Sepsityper kit® (Bruker Daltonics)			75.6% (n = 160)	35 min (5 centrifugation steps)
[45]				80.8% (n = 411)	
[30]				79.8% (n = 3320)	
[46]	Vitek MS Blood culture kit® (Biomérieux)			73% (n = 259)	15 min (No centrifugation steps)
[47]	Rapid BACpro®II kit (Nittobo Medical Co)			76.5% (n = 17)	15 min (4 centrifugation steps)
[43]	Rapid BACpro®II kit (Nittobo Medical Co) improvement			96,3% (n = 269)	
This study	Rapid Sepsityper® protocol (Bruker Daltonics): RS ± FA Complete Sepsityper® protocol on unidentified samples (n = 94)			68.6% (n = 299) 78.6% (n = 299)	10 min (2 centrifugation steps) 35 min (5 centrifugation steps)
***In-house protocols: centrifugation***					
[48]	Centrifugation			95% (n = 277)	> 20 min (> 5 centrifugation steps)
[41]	Centrifugation			43% (n = 79)	> 10 min (4 centrifugation steps)
[49]	Centrifugation			85.9% (n = 85)	15 min (2 centrifugation steps)
***In-house protocols: centrifugation in Separator tube***					
[50]	Clot activator and gel BD Vacutainer tubes® (BD Diagnostics) ACUETTE® Z Serum Sept Clot Activator (Greiner Bio-One)			89.6% (n = 532)	> 20 min (5 centrifugation steps)
[51]	ACUETTE® Z Serum Sept Clot Activator (Greiner Bio-One)			90% (n = 186)	15 min (2 centrifugation steps)
[42]	Separator tube with plasma separation gel			88.7% (n = 789)	> 20 min (2 centrifugation steps)
[52]	Serum Separator tube (BD Diagnostics)			68.7% (n = 195)	> 30 min (5 centrifugation steps)
***In-house protocols: centrifugation + lysis reagent***					
[53]	5% Saponin lysis (fast protocol) 20% SDS lysis (fast protocol)			53% (n = 42) 86% (n = 42)	20 min (2 centrifugation steps)
[54]	0.6% polyoxyethylene 10 oleoyl ether (Brij 97) in 0.4 mol/L 3-cyclohexylamino-1-propane sulfonic acid lysis			82,4% (n = 125)	> 10 min (2 centrifugation steps)
[34]	Triton X-100 lysis			80.5% (n = 681)	10 min (2 centrifugation steps)
***In-house protocols: Centrifugation + lysis reagent + additional protein extraction***					
[55]	Saponin lysis + 10% TFA extraction			61.4% (n = 127)	20 min (2 centrifugation steps)
[56]	5% Saponin lysis + Ethanol/FA extraction			80.6% (n = 155)	20 min (2 centrifugation steps)
[57]	5% Saponin lysis 10% SDS lysis + 100% Ethanol/100% Acetonitrile/70% FA extraction			79.5% (n = 176) 88% (n = 176)	> 20 min (5 centrifugation steps)
[41]	10% SDS lysis + 70% FA/Acetonitrile extraction			49% (n = 101)	> 20 min (4 centrifugation steps)
[58]	Ammonium chloride lysis + 70% Ethanol/70% FA extraction			78.7% (n = 122)	30–45 min (4 centrifugation steps)
[59]	0.1% TFA lysis + 70% FA/100% Acetonitrile extraction			88.9% (n = 245)	> 40 min (6 centrifugation steps)
[40]	0.2% Triton X-100 + 0.1% SDS lysis 0.2% Triton X-100 + 1.8% SDS lysis + Ethanol/100% Acetonitrile/70% FA extraction + 70% FA extraction			86.3% (n = 450) 86.4% (n = 59)	30 min (5 centrifugation steps) 10 min (3 centrifugation steps)

The technical time is either directly issued from publications when available or estimated from the number of centrifugation and centrifugation time described in protocols (estimated as > to x min with centrifugation steps between 1 to 3 min). (SDS: Sodium dodecyl sulfate; FA, formic acid; TFA, trifluoroacetic acid; n, number of samples tested)

In this study, we prospectively evaluated the microbiological performances of a new rapid protocol using the Sepsityper® kit and of the specific MBT-Sepsityper module. This rapid protocol allows bacterial and yeast identification directly from positive blood cultures within 10 min of turnaround time while MBT-Sepsityper module has been designed to increase identification performance compared to standard MBT-Compass-IVD module. To provide an optimized diagnosis strategy we also evaluated the benefit of using an on-plate formic acid extraction step and compared identification rates depending on the type of positive blood culture bottles (aerobic or anaerobic) for facultative anaerobes.

## Materials & methods

### Sample collection

**Fig. 1:**
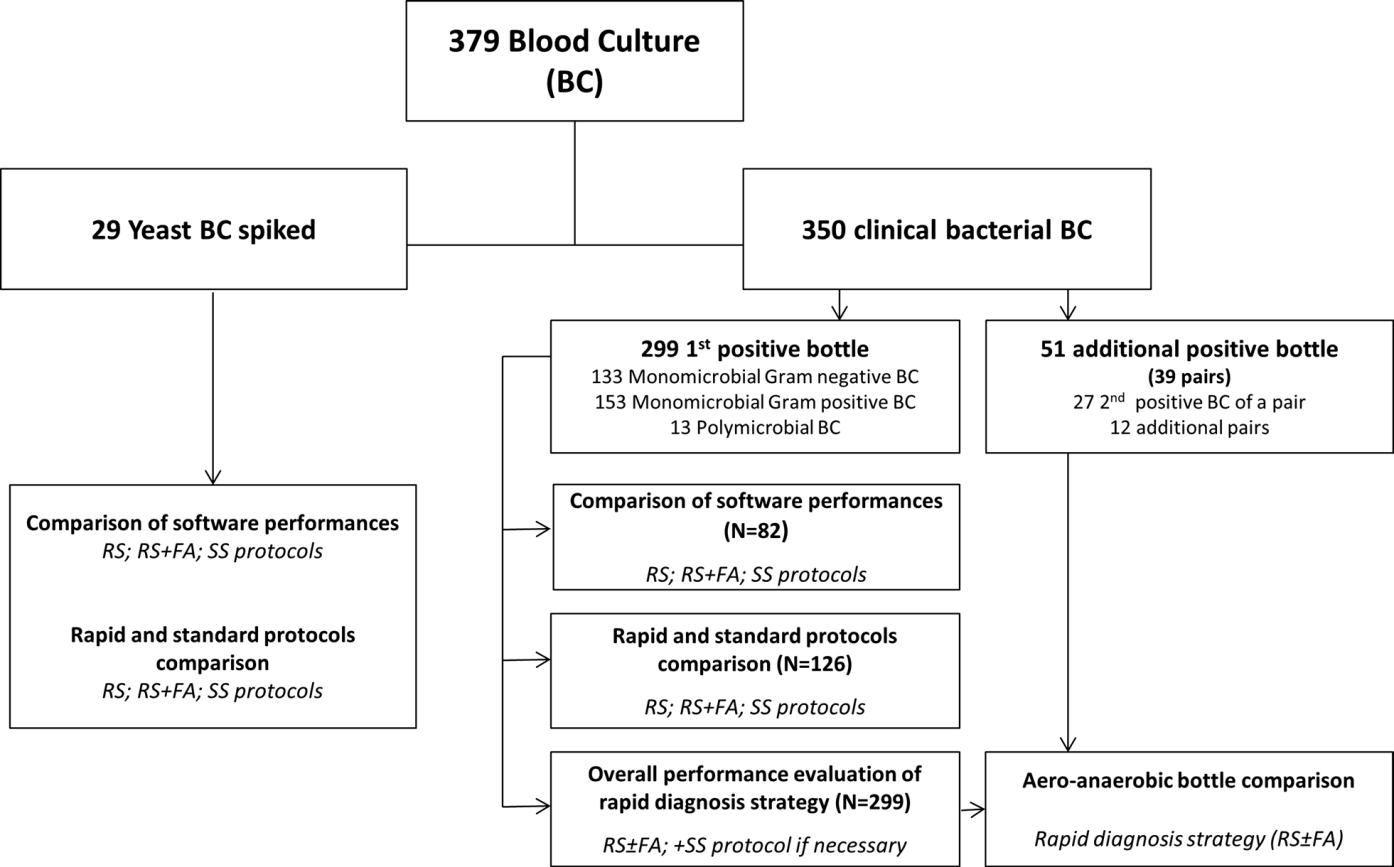
Flowchart of the study design. RS, Rapid Sepsityper® protocol; RS + FA, Rapid Sepsityper® protocol with formic acid extraction step; RS ± FA, Rapid Sepsityper® protocol with and without formic acid step (Rapid diagnosis strategy), SS, Standard Sepsityper®. (N, number of samples tested; BC, blood culture)

Three hundred and seventy-nine positive blood cultures bottles were analyzed (143 aerobic: BACTEC Plus Aerobic/F, 182 anaerobic: BACTEC Lytic/10 Anaerobic/F, 25 pediatric: BACTEC Peds Plus/F, and 29 BACTEC Mycosis IC/F; Becton Dickinson) (Fig. 1). All the blood cultures were incubated in a BD BACTEC™ FX instrument (Becton Dickinson). Three hundred and fifty positive blood culture growing bacteria have been collected from patients hospitalized at Grenoble Alpes University Hospital between June 2017 and July 2018 and were analyzed prospectively within 12 h of positivity of the blood culture bottle. Among those, two hundred and ninety-nine samples have been selected as they corresponded to the first positive blood culture bottle for all new episodes of bacteremia during random days. Fifty-one positive blood cultures bottles were also included in the study in order to compare identification rates obtained on aerobic or anaerobic bottles for facultative anaerobes (27 second positive bottle of a pair of blood culture already included and 12 additional pairs of positive blood culture). Because fungemia was uncommon, and to obtained an important strain diversity, the twenty-nine BACTEC Mycosis IC/F blood culture were artificially spiked with different species of yeast from frozen laboratory strains: *C. albicans* (5); *C. glabrata* (3); *C. kefyr* (3); *C. dubliniensis* (2); *C. parapsilosis* (2); *C. guillermondii* (2); *C. norvegiensis* (2); *C. krusei* (2); *C. lusitaniae* (1); *C. tropicalis* (1); *C. orthopsilosis* (1); *C. anomalus* (1); *Cryptococcus neoformans* (2) and *S. cerevisiae* (2). An average of 10 CFU was inoculated into the blood culture bottle previously filled with 7 to 10 ml of healthy volunteers' blood, following the methodology of a previously published protocol [60].

### Reference protocol

Reference identification protocol used in the bacteriology and mycology laboratories of Grenoble Alpes University Hospital was used as gold standard of identification for the study. In brief, positive blood cultures were subcultured on various agar medium based on the results of the Gram stain, and MALDI-TOF-MS identification was performed on colony after 14–48 h of incubation depending on the growth speed of the bacteria/yeast. Identification mass spectra were acquired on a Microflex LT MALDI-TOF (Bruker Daltonics) and analyzed using MBT-Compass-IVD database (DB-7171 v.7.0.0.0). Identification to the species level on bacterial colonies was considered reliable if the identification score was ≥ 2 or if the score was between 1.8 and 2 with the five best matches belonging to the same species and with characteristics in accordance with all the other available data (Gram stain, catalase, coagulase, oxidase…).

### Rapid and standard Sepsityper® protocols

**Fig. 2:**
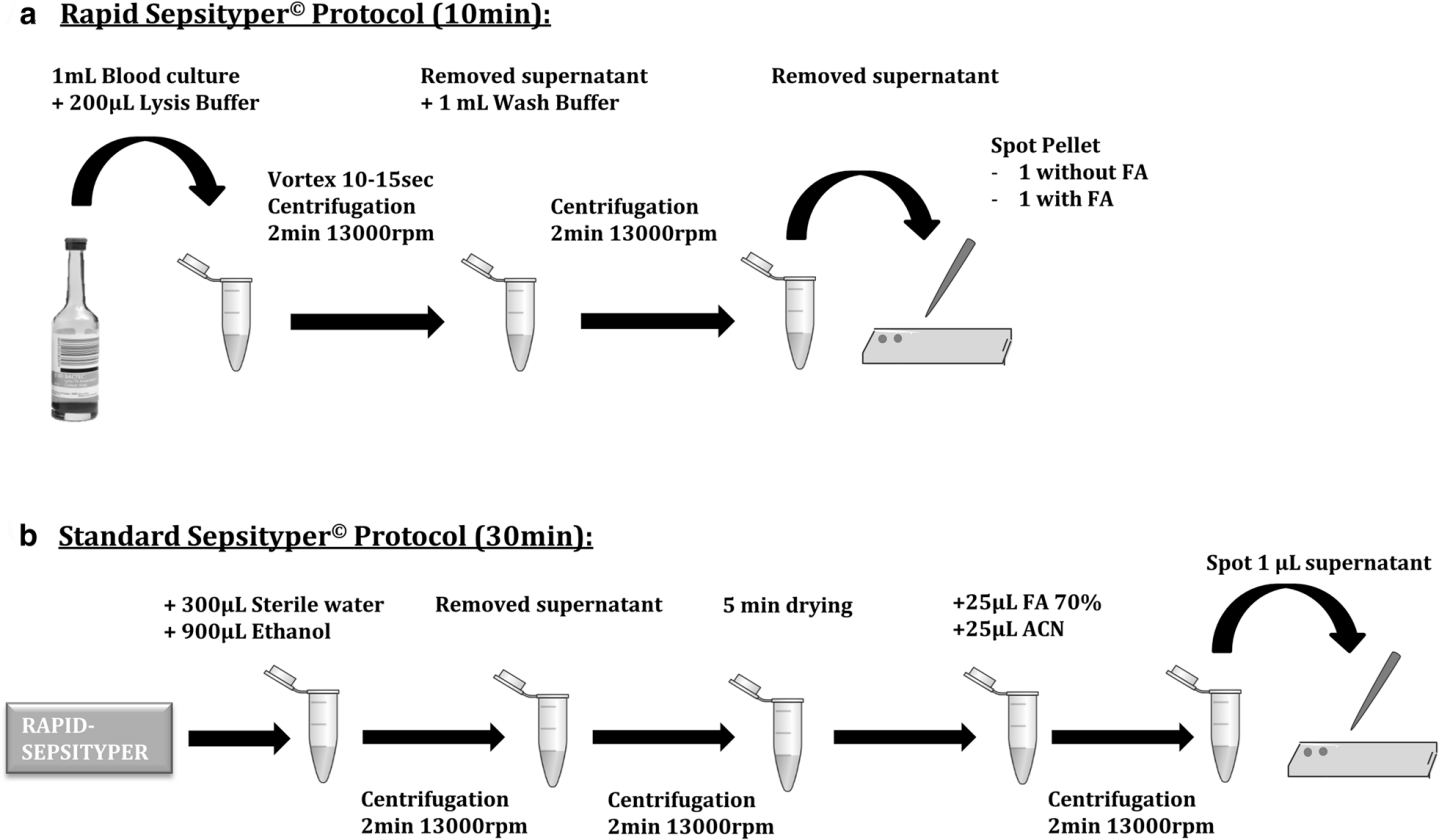
Technical workflow of the Rapid Sepsityper® protocol (**a**) and the Standard Sepsityper® protocol (**b**). Standard Sepsityper protocol requires to initially perform the Rapid Sepsityper protocol and to continue with steps presented in part B of the Figure. (FA: Formic Acid; ACN: Acetonitrile; rpm: rounds per minute)

MALDI-TOF-MS identification was performed using either the Rapid protocol (10 min turnaround time) of the Sepsityper® kit or the Standard procedure (30 min turnaround time) according to the manufacturer’s recommendations. In brief, for the Rapid Sepsityper® (RS) protocol, 1 mL of positive blood culture was transferred to an Eppendorf tube and 200 µL of lysis buffer were added. The sample was vortexed 10–15 s then centrifuged 2 min at 13000 rpm. Supernatant was discarded and the pellet re-suspended in 1 mL of washing buffer and centrifuged again 1 min at 13000 rpm. Supernatant was removed and 1µL of pellet was spotted on a MALDI-TOF target. For each sample, 2 spots were filled and analyzed: one without and one with on-plate addition of 1 µL of 70% formic acid (FA) before drying and addition of 1µL of the matrix (IVD Matrix, HCCA-portioned, Bruker Daltonics) to all the spots (defined respectively as RS and RS + FA protocols) (Fig. 2a). Protocol was then continued with the Standard Sepsityper procedure (defined as SS protocol). The remaining pellet was re-suspended with 300µL of sterile water and 900µL of absolute ethanol. The sample was centrifuged 2 min at 13000 rpm, the supernatant removed and a second centrifugation step was performed. Residual ethanol was air dried five minutes. Then an equal amount of formic acid and acetonitrile (ACN) were added and the sample was centrifuged 2 min at 1300 rpm. Finally, 1µL of supernatant was spotted on the MALDI-TOF target (Fig. 2b).

MALDI‑TOF-MS data analysis and evaluation of performance were all acquired on a Microflex LT MALDI-TOF (Bruker Daltonics) mass spectrometer. The study was conducted in three steps. First, the comparison of spectrum analysis performances of MBT-Compass-IVD v.7.0.0.0 (DB-7171) and MBT-Sepsityper-RUO v.7 (DB-7311) (specific software for analysis of positive blood cultures) was assessed on the 82 first positive bacterial samples and the 29 artificial yeast samples. Using MBT-Compass-IVD, a green score (≥ 2), a yellow score [1.7–1.99] and a red score (< 1.69) corresponded respectively to an identification to the species level with high reliability, with low reliability and to no identification. Using MBT-Sepsityper-RUO, the algorithm defined by the manufacturer sets lower cut-offs to obtain a green, yellow or red score: ≥ 1.8, [1.6–1.79] and < 1.59 respectively. Thus, in our study, identification to the species level was considered reliable with MBT-Sepsityper-RUO module if the identification score was ≥ 1.6 and matched the identification obtained with reference protocol.

Secondly identification performance to the species level obtained with the RS or RS + FA protocols versus SS protocol were compared on the first 126 positive blood cultures growing bacteria and on the 29 spiked fungal samples using only the MBT-Sepsityper-RUO software (as step one showed significantly more reliable identification with this software). Thirdly, overall performance of a rapid diagnosis strategy using RS protocol on one spot and RS + FA protocol on a second spot (defined as RS ± FA) with MBT-Sepsityper-RUO software analysis (as step two showed high performances of this strategy) was assessed for 299 positive blood cultures growing bacteria. In case of failure of the RS ± FA protocol to provide a reliable identification, additional steps to fulfill the SS protocol were performed on the remaining pellet. Finally, to provide an optimized workflow, we also compared identification rates obtained with the rapid diagnosis strategy (RS ± FA) on aerobic and anaerobic bottles of the 299 first positive blood culture and confirm observed results on 39 pairs of positive blood cultures growing facultative anaerobes bacteria.

### Statistical analysis

All statistical analyses were performed on R Studio software (version 3.6.0) using Mac Nemar test (paired Chi^2^-test) with continuity correction or χ2 test when appropriate. A p-value < 0.05 was considered significant.

## Results

This study was a non-interventional study evaluating microbiological performances of the Rapid Sepsityper® RUO protocol and of specific MBT-Sepsityper-RUO module without real-time result transmission to clinicians.

### Comparison of MBT-Sepsityper-RUO and MBT-Compass-IVD software performances

**Fig. 3:**
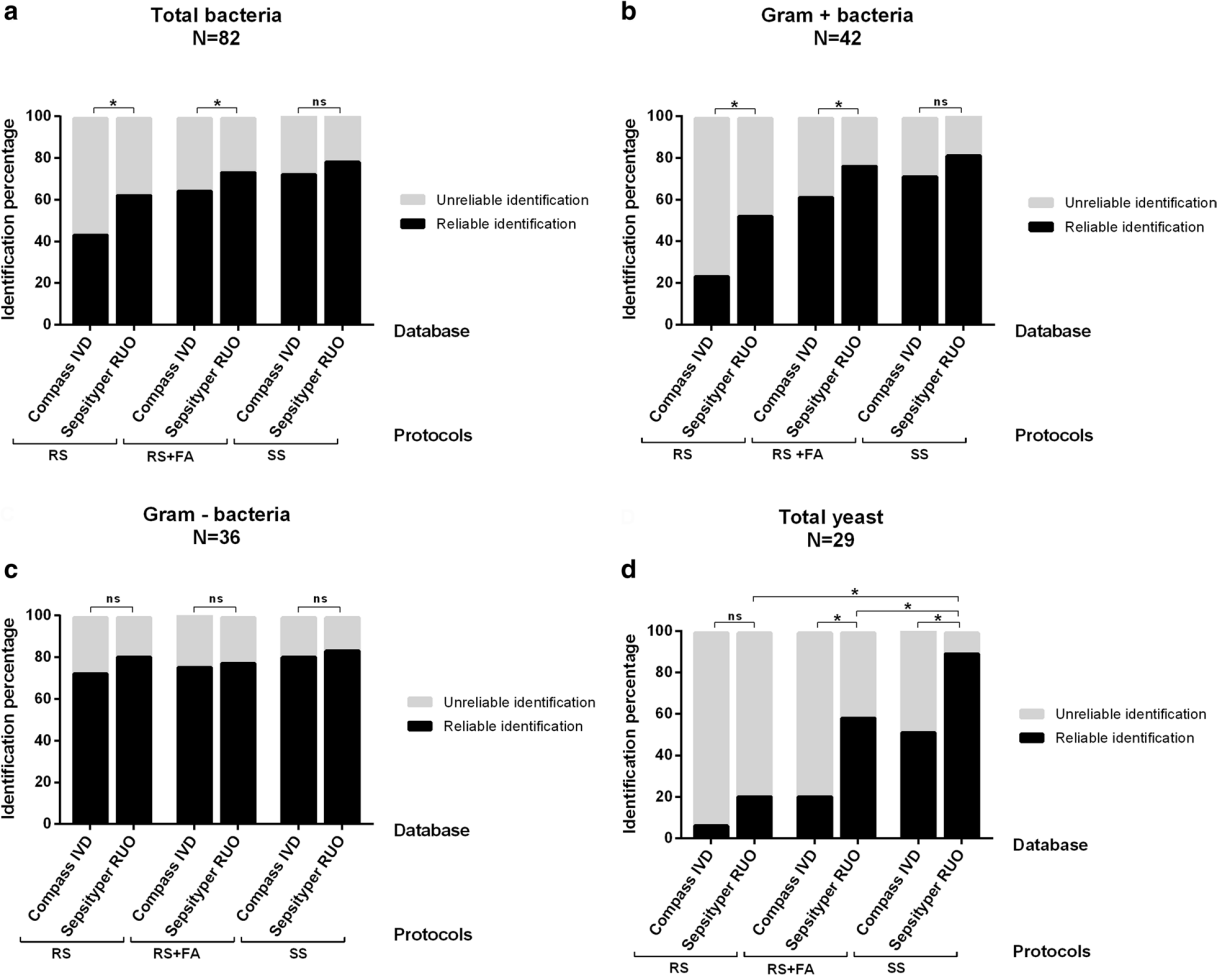
Comparison of the performances of standard MBT-Compass-IVD database and specific MBT-Sepsityper-RUO database. Percentage of reliable and unreliable identification using Compass-IVD or Sepsityper-RUO database for all monomicrobial and polymicrobial bacterial blood cultures analyzed (**a**), for monomicrobial positive blood cultures with Gram positive bacteria (**b**), for monomicrobial positive blood cultures with Gram negative bacteria (**c**) and for monomicrobial positive blood cultures with yeast (**d**). Results are also stratified according to the use of either the Rapid Sepsityper® (RS), Rapid Sepsityper® with formic acid extraction step (RS + FA) or Standard Sepsityper® (SS) protocols.(N: number of samples tested in each group; ns: no statistically significant difference; *: p < 0.05)

Performances of both software were compared on 111 positive blood cultures (42 GP, 36 GN, 4 polymicrobial samples and 29 yeast). Each blood culture was treated using RS, RS + FA and SS protocols. Percentage of reliable identification to the species level obtained for each protocol and each software are represented in Fig. 3. MBT-Sepsityper-RUO provided significantly higher percentage of reliable identification than MBT-Compass-IVD with both RS (62.2% (51/82) vs 43.9% (36/82), p = 0.001) and RS + FA protocols (73.2% (60/82) vs 64.6% (53/82), p = 0.023) but only a tendency of higher identification was observed for the SS protocol (78% (64/82) vs 72% (59/82), p = 0.074) (Fig. 3a). Stratification according to Gram stain revealed that MBT-Sepsityper-RUO provided higher reliable identification percentage only for monomicrobial GP bacteremia, with both RS (52.4% (22/42) vs 23.8% (10/42), p = 0.006) and RS + FA protocols (76.2% (32/42) vs 61.9% (26/42), p = 0.041) (Fig. 3b). No significant difference was found for RS, RS + FA or SS protocols for monomicrobial GN bacteremia: 80.6% (29/36) vs 72.2% (26/36) (p = 0.248), 77.8% (28/36) vs 75% (27/36) (p = 1) and 83.3% (30/36) vs 80.6% (29/36) (p = 1) respectively (Fig. 3c). All mycosis positive blood culture combined, MBT-Sepsityper-RUO module provided significantly higher percentage of reliable identification level than MBT-Compass-IVD module with both RS + FA (58.6% (17/29) vs 20.7% (6/29), p = 0.003) and SS protocol (89.7% (26/29) vs 51.7% (15/29), p = 0.003). With RS protocol however, reliable identification rates were lower than 21% with no significant difference between MBT-Sepsityper-RUO and MBT-Compass-IVD modules (20.7% (6/29) vs 6.9% (2/29) respectively, p = 0.134) (Fig. 3d).

Thus we selected MBT-Sepsityper-RUO software for all further experiments.

### Comparison of rapid Sepsityper® and Standard Sepsityper® protocols performances

**Fig. 4:**
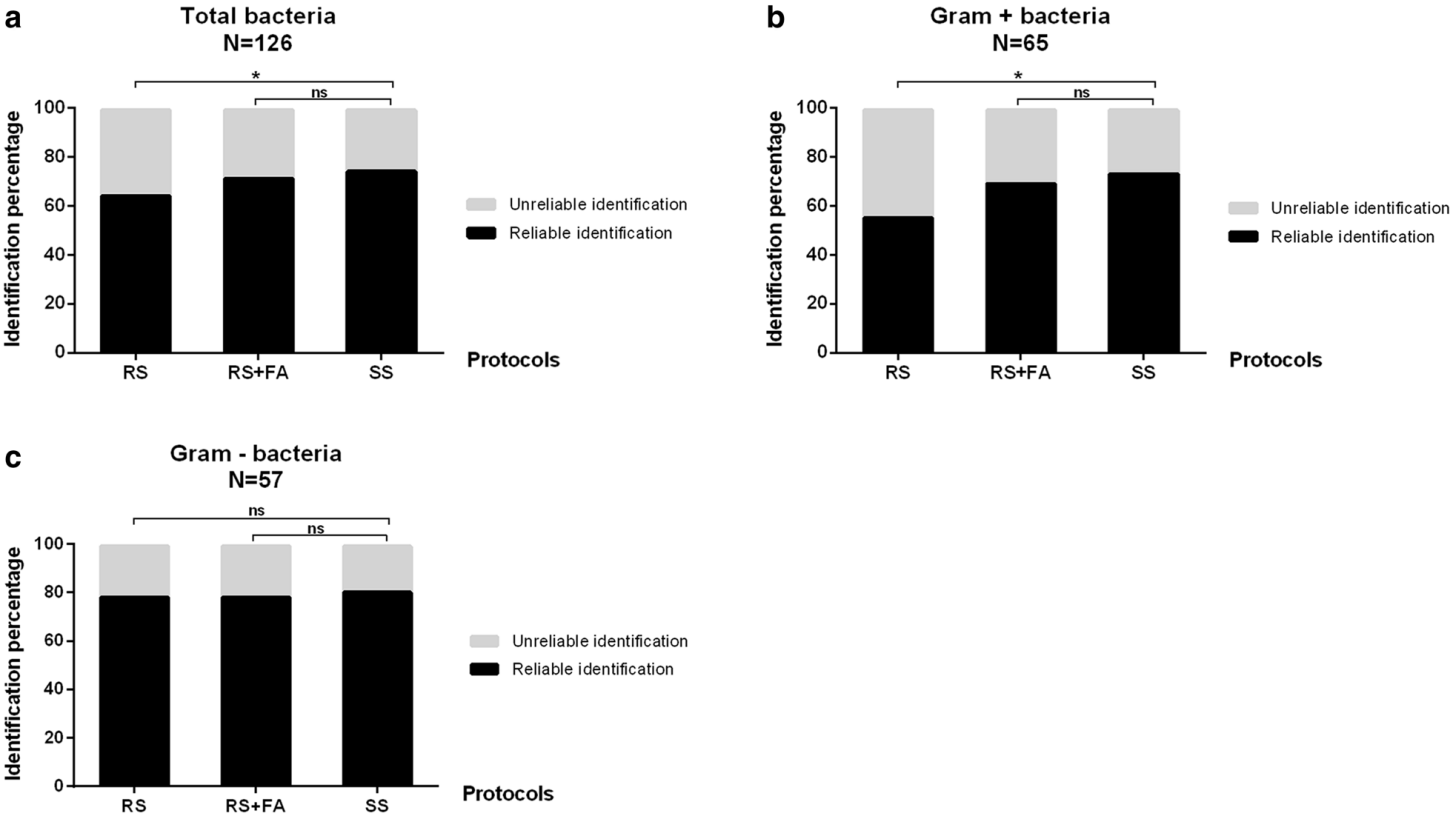
Comparison of the performances of Rapid and Standard Sepsityper protocols on bacterial positive blood cultures. Percentage of reliable and unreliable bacterial identification with either the Rapid Sepsityper® (RS), Rapid Sepsityper® with formic acid extraction step (RS + FA) or Standard Sepsityper® (SS) protocols for all monomicrobial and polymicrobial positive blood cultures (**a**), for monomicrobial positive blood cultures with Gram positive bacteria (**b**), and for monomicrobial positive blood cultures with Gram negative bacteria (**c**). (N: number of samples tested in each group; ns: no statistically significant difference; *: p < 0.05)

Bacterial identification rates of RS, RS + FA and SS protocol were compared on 126 positive blood cultures (65 GP, 57 GN and 4 polymicrobial samples). For all positive blood culture combined, reliable identification rates were 64.3% (81/126), 71.4% (90/126) and 74.6% (94/126) with RS, RS + FA and SS protocols respectively. For monomicrobial GP bacteremia, reliable identification rates were 55.4% (36/65), 69.2% (45/65) and 73.8% (48/65) with RS, RS + FA and SS protocols respectively while they were 78.9% (45/57), 78.9% (45/57) and 80.7% (46/57) respectively for monomicrobial GN bacteremia (Fig. 4). Thus performances of RS + FA protocol did not differ significantly from those of SS protocol (p = 0.45, p = 0.55 and p = 1 for all positive blood culture combined, monomicrobial GP and GN bacteremia respectively) while providing faster results. Stratification by Gram stain showed however that RS protocol was less performing than SS protocol for identification of GP bacteria (p = 0.02, p = 0.03 and p = 1 for all positive blood culture combined, monomicrobial GP and GN bacteremia respectively) (Fig. 4). As regards the 4 polymicrobial blood cultures, only one species on the two present was accurately identified, with no warning highlighting that the blood culture may be polymicrobial.

Performances on yeast identification of the three protocols were compared on 29 artificial positive blood cultures (Fig. 3d). SS protocol provided 89.7% (26/29) reliable identification while RS and RS + FA protocols were significantly less efficient with only 20.7% (6/29) (p = 2.2e^−5^), and 58.6% (17/29) (p = 0.008) reliable identification rates, respectively. Performance of the rapid diagnosis strategy (RS ± FA) with MBT-Sepsityper-RUO software analysis provided 62% (18/29) reliable identification.

**Fig. 5:**
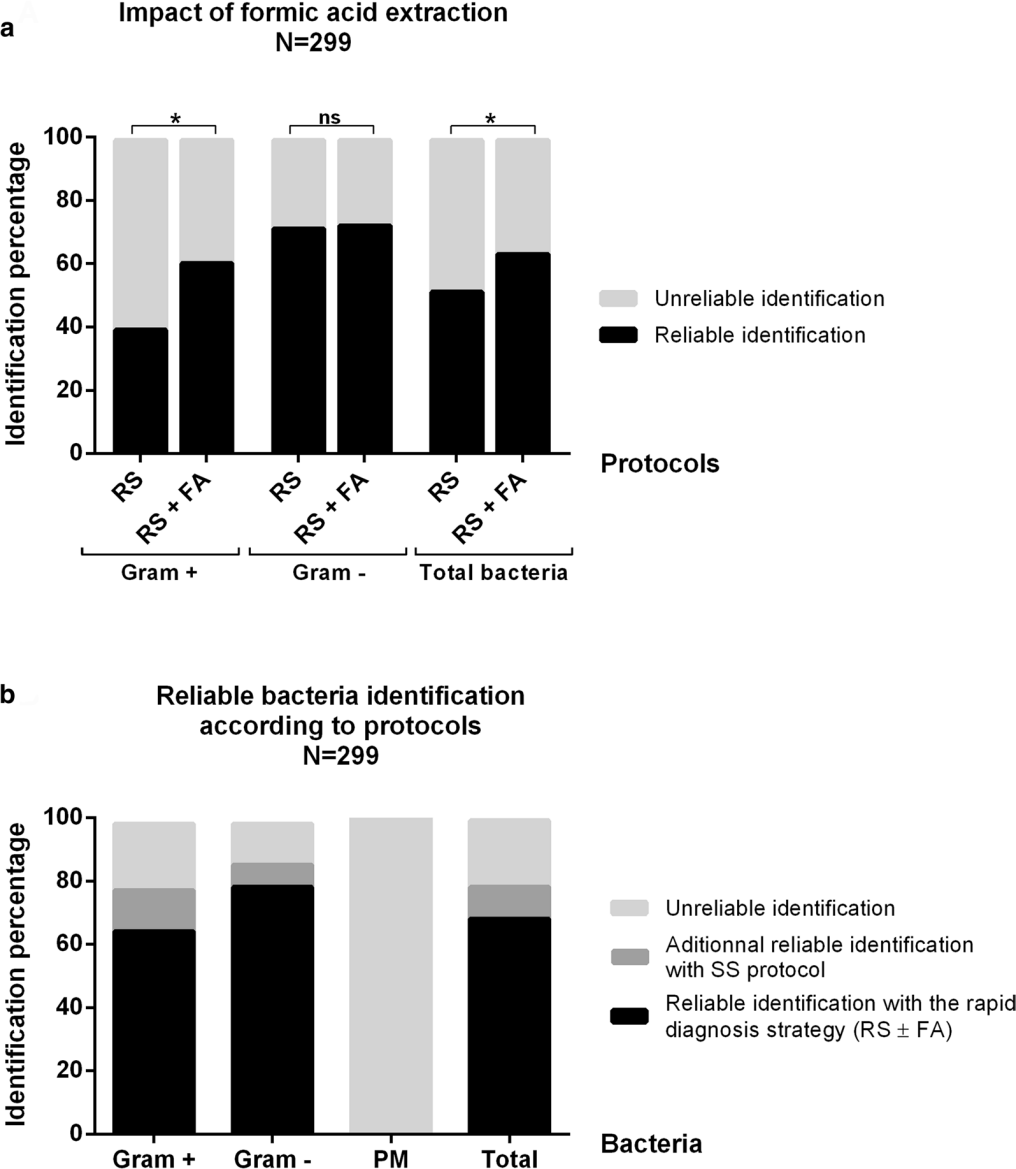
Impact of acid formic extraction step and performances of our rapid diagnosis strategy. **a** Percentage of reliable and unreliable bacterial identification with either the Rapid Sepsityper® (RS) or the Rapid Sepsityper® with formic acid extraction step (RS + FA) protocols for all monomicrobial and polymicrobial positive blood cultures (Total bacteria), monomicrobial positive blood cultures with Gram positive (Gram +) and monomicrobial positive blood cultures with Gram negative bacteria (Gram −). (N: number of samples tested; ns: no statistically significant difference; *: p < 0.05). **b** Percentage of reliable and unreliable bacterial identification obtained with the rapid diagnosis strategy (Rapid Sepsityper® with and without formic acid protocols: RS ± FA) or after Standard Sepsityper® (SS) completion (for samples unidentified with the rapid protocol) for all monomicrobial and polymicrobial positive blood cultures (Total), for monomicrobial positive blood cultures with Gram positive bacteria (Gram +), for monomicrobial positive blood cultures with Gram negative bacteria (Gram −) and for polymicrobial positive blood cultures (PM)

Finally, we compared on a larger number of samples (299 first bacterial positive blood cultures) the performances of RS versus RS + FA protocols to measure the impact of on-plate formic acid extraction step. We also assessed global performances of a rapid diagnosis strategy (RS ± FA) with analysis of the spectra thanks to MBT-Sepsityper-RUO software. RS + FA protocol showed significantly higher identification rates than RS (63.5% (190/299) vs 51.8% (155/299), p = 1.8e^−5^). Stratification according to Gram stain results showed that formic acid improved significantly the identification rates for monomicrobial GP bacteremia: 60.8% (93/153) vs 39.2% (60/153) (p = 1.8e^−6^) but not for GN bacteremia: 72.9% (97/133) vs 71.4% (95/133) (p = 0.814) (Fig. 5a). Overall, a rapid diagnosis strategy with MALDI-TOF–MS analysis of two spots (RS ± FA) allowed 68.6% (205/299) reliable identification to the species level. Completion of SS protocol in case of unreliable results increased identification percentage by 10% (236/299), while 21.1% of positive blood cultures remained unidentified. Stratification by Gram stain revealed that this rapid strategy provided 65.4% and 78.9% reliable identification for monomicrobial GP and GN bacteremia respectively. SS protocol completion for unidentified samples increased those rates to 79.1% for GP bacteria and to 86.4% for GN bacteria. Among the 13 polymicrobial samples, one species was identified in 9/13 (69%) but the software failed to detect that any samples was polymicrobial whatever the protocol used (Fig. 5b).

### Impact of the type of blood culture bottle

Identification rates of bacteria obtained on positive aerobic or anaerobic bottles were compared on the 299 unpaired positive blood culture included in the study. Results showed 82.9% (58/70) vs 48.5% (32/66) reliable identification of GP bacteria (p < 0.05) and 85.1% (63/74) vs 69.8% (37/53) reliable identification of GN bacteria (p < 0.05) in anaerobic versus aerobic bottle respectively using the rapid strategy (RS ± FA). We later confirmed this tendency on thirty-nine pairs of positive blood culture bottles. Reliable identification rate was significantly higher in anaerobic bottles compared to aerobic bottles: 87.2% (34/39) and 56.4% (22/39) respectively (p = 0.003) (Additional file 1: Figure S1). Stratification by Gram stain showed no significant difference for monomicrobial GN bacteremia with 95.2% (20/21) and 85.7% (18/21) identification rates in anaerobic and aerobic bottle respectively (p = 0.8) but the number of sample analyzed was low. However, for monomicrobial GP bacteremia, identification rates were significantly higher in anaerobic bottle than in aerobic bottle: 77.8% (14/18) vs 22.2% (4/18) (p = 0.004) respectively.

### Sensitivity of the rapid strategy

On the 205 monomicrobial positive blood culture reliably identified with the rapid strategy, 105 (51.2%) were positive with GN bacteria and 100 (48.8%) with GP bacteria. The sensitivity of the rapid strategy for each bacterial species is represented in Table 2. Among unidentified bacteria, GP bacteria were predominant over GN bacteria (53 vs 28). Coagulase-negative *Staphylococcus* was the main GP genus unidentified followed by *Streptococcus*: 20 (37.7%) and 16 strains (30.2%) respectively, while *Enterobacterales* and anaerobic bacteria were the most unidentified GN bacteria: 12 (42.8%) and 7 (25%) respectively.

**Table 2:** Bacterial identification results and sensitivity of detection of the rapid diagnosis strategy

Bacterial species	Sensitivity (%)	Bacterial species	Sensitivity (%)
Gram positive bacteria		Gram negative bacteria	
***Staphylococcus* (n = 98)**		***Enterobacterales*** **(n = 109)**	
*S. aureus*	30/37 (81.1%)	*E. coli*	72/77 (93.5%)
***CoNS***		*K. pneumoniae*	6/7 (85.7%)
*S. epidermidis*	28/43 (65.1%)	*P. mirabilis*	6/7 (85.7%)
*S. hominis*	8/9 (88.9%)	*E. complex cloacae*	5/5 (100%)
*S. haemolyticus*	2/3 (66.7%)	*C. freundii*	2/2 (100%)
*S. capitis*	2/2 (100%)	*C. koseri*	2/2 (100%)
*S. lugdunensis*	1/1 (100%)	*H. alvei*	2/2 (100%)
*S. pasteuri*	0/1 (0%)	*S. marcescens*	1/2 (50%)
*S. caprae*	0/1 (0%)	*K. variicola*	0/2 (0%)
*S. pettenkoferi*	0/1 (0%)	*K. oxytoca*	0/1 (0%)
***Streptococcus*** **(n = 32)**		*Salmonella sp*	1/1 (100%)
*S. pneumoniae*	1/6 (16.7%)	*P. agglomerans*	0/1 (0%)
*S. gallolytcus*	3/6 (50%)	***Others *(n = 24)**	
*S. mitis/oralis*	3/5 (60%)	*P. aeruginosa*	7/8 (87.5%)
*S. pyogenes*	3/4 (75%)	*H. influenzae*	0/2 (0%)
*S. agalactiae*	2/2 (100%)	*F. nucleatum*	0/2 (0%)
*S. milleri group*	2/3 (66.7%)	*B. fragilis*	0/2 (0%)
*S. dysgalactiae*	2/2 (100%)	*R. radiobacter*	1/1 (100%)
*S. gordonii*	0/2 (0%)	*L. trevisani*	0/1 (0%)
*S. parasanguinis*	0/1 (0%)	*P. heparinolytica*	0/1 (0%)
*S. sanguinis*	0/1 (0%)	*B. uniformis*	0/1 (0%)
***Enterococcus*** **(n = 11)**		*C. jejuni*	0/1 (0%)
*E. faecalis*	5/6 (83.3%)	*C. fetus*	0/1 (0%)
*E. faecium*	4/5 (80%)	*O. anthropi*	0/1 (0%)
***Others*** **(n = 12)**		*A. caviae*	0/1 (0%)
*C. acnes*	1/2 (50%)	*A. ursingii*	0/1 (0%)
*F. magna*	1/1 (100%)	*R. mucosa*	0/1 (0%)
*M. luteus*	1/1 (100%)		
*L. rhamnosus*	1/1 (100%)		
*R. dentocariosa*	0/1 (0%)		
*L. casei*	0/1 (0%)		
*P. micra*	0/1 (0%)		
*C. ramosum*	0/1 (0%)		
*C. minutissimum*	0/1 (0%)		
*G. aichiensis*	0/1 (0%)		
*Microbacterium sp*	0/1 (0%)		
Total	100/153 (65.4%)		105/133 (78.9%)

Bacterial species identified by the rapid diagnosis strategy (RS ± FA) in Gram-positive and Gram-negative monomicrobial positive blood culture

## Discussion

While blood culture remains the reference method for BSI diagnosis, new rapid methods have emerged to reduce the delay to identify bacterial and fungal pathogens. They may be realized directly from whole blood (multiplex PCR; T2 magnetic resonance) [61–65] but are mainly performed on positive blood culture bottles using either MALDI-TOF-MS-based techniques (direct identification after purification of bacterial or fungal pellets; identification after short-term incubation on a solid medium) [30–33, 45] or molecular-based techniques (multiplex-PCR, PNA-FISH) [31, 35–37, 39].

Reliable bacterial identification rates obtained with molecular-based techniques currently range between 82.5% to 96% [35–37, 39, 66] and reliable fungal identification from 23 to 100% [31, 37, 66–69] in 20 to 90 min. Multiplex PCR panels can also detect important antibiotic resistance genes. They have however still a relatively high cost (100–300€/cartridge) and identification is restricted to a limited number of species included in the assays (11 to 35 bacterial species and up to 15 yeast species). On the other hand, MALDI-TOF–MS offers solutions at reduced costs but with a higher turnaround time (Table 1). The majority of direct identification protocols are “in-house” protocols using variable extraction steps and lysis reagents (Table 1). Centrifugation alone with no lysis reagent has shown sensibilities ranging from 43 to 95% between studies and can be performed using a separator tube [41, 42, 48–52]. Different lysis reagents may be employed, in particular Saponin, Sodium dodecyl sulfate (SDS), Ammonium chloride or Triton X-100 with sensibilities ranging from 53 to 86% [34, 53, 54]. Additional extraction steps using either ethanol, formic acid or acetonitrile have also been described improving sensibilities from 49% to 88.9% [40, 41, 55–59]. Time to results relies mainly on the number of extraction and centrifugation steps in each protocol and therefore varies between 10 to 45 min which limits the use of many of them in routine diagnostic procedures (Table 1) [34, 40, 41, 53–59]. Thus, any improvement and reduction of turnaround time in MALDI-TOF–MS protocols allowing more laboratories to perform such rapid identification is of great clinical importance. Moreover, in-house protocols may have a lack of standardization, often use modified cut-offs not validated by the MALDI-TOF-MS manufacturer and require on site validation for certification [34, 57]. A few commercial kits have been developed. The Vitek MS Blood Culture kit® (BioMérieux) uses a filtration-based method and the Rapid BACpro®II (NIttobo Medical Co) a copolymerization method for bacterial extraction and allow respectively 73% and between 76.5% to 96.3% reliable bacterial identification in 15 min [43, 46, 47]. The Sepsityper® kit is another CE-IVD commercial assay. According to a review and meta-analysis of its performance, it allows identification in 30 min of 79.8% of bacterial samples to the species level (76.1% and 89.6% for monomicrobial GP and GN bacteremia respectively) while fungal reliable identification rate is 65.9% [30]. However, its important technical turnaround time, due to at least 5 centrifugation steps, also limits its integration in routine procedures [28].

In this study we evaluated the rapid version of the Sepsityper® protocol that reduces hands-on time and identification delay to 10 min as it only requires one lysing step, one washing step and 2 centrifugation steps. Moreover, we assessed the impact of the use of specific MBT-Sepsityper module dedicated to the analysis of positive blood culture which indeed significantly increased reliable bacterial identification rate by 18.3% and 8.6% for RS and RS + FA protocol respectively compared to standard MBT-Compass-IVD database. With the use of MBT-Sepsityper module we observed that the rapid strategy (RS ± FA) seemed to be as effective as the SS protocol with bacterial identification rates of 78.9%, 65.4% and 68.6% for monomicrobial GN, GP and all bacteremia respectively while dividing turnaround time by three. Overall, our rapid strategy had between 5 to 25% less sensitivity than several studies summarized in Table 1, but these other protocols either require more hands-on time (up to 45 min) or use modified MALDI-TOF cut-offs not validated by the manufacturer. Our rapid strategy provided results in 10 min for 2/3 of the samples and in case of failure, was continued with the SS protocol which allowed 10% additional identification while being done only on 1/3 of the samples, thus reducing global hands-on time.

Formic acid use was mandatory to increase identification rate of GP bacteria which otherwise dropped from 20% between RS + FA and RS protocols but remained lower than for GN bacteria, as already experienced in other studies [30, 35]. It may be explained by the adherence of GP bacteria to the red blood cells and their removal with the serum (P. Murray, Becton Dickinson, personal communication), by a more robust cell wall which decreases protein extraction yields or by a smaller pellet after extraction due to a slower growth or lower biomass of GP bacteria in positive blood cultures [30, 52]. Indeed, a bacterial load inferior to 10^6^ CFU/mL has been proved to give MALDI-TOF-MS spectrum peaks indistinguishable from background peaks [45]. Interestingly, significantly higher identification rates were observed when analyzing BD BACTEC anaerobic positive blood culture compared to aerobic bottles for GP bacteria. The presence of small resin beads in the BD BACTEC Aerobic bottles may interfere with protein extraction and thus it may not be the case with all blood culture bottles manufacturers. On the other hand, the presence, of a lytic reagent (saponin) in BD BACTEC Anaerobic bottles may enhance bacterial identification by weakening bacterial cell wall or by improving red blood cells lysis.

As regards yeast identification, SS protocol appeared to be more effective than rapid RS or RS + FA protocols even though formic acid also improved performances. Importantly, the use of specific MBT-Sepsityper module had a very significant impact on yeast identification rate as it increased identification rates by 38% for RS + FA and SS protocols. While in previous studies SS results ranged from 56% to 65.9% reliable identification rates using MBT-Compass-IVD database, we obtained similar identifications rates while requiring only 10 min turnaround time with the RS + FA protocol (58.6%) when using the MBT-Sepsityper module and significantly higher results with the SS protocol and the MBT-Sepsityper module (89.7%) [30, 70–73]. Some limitations of our study have to be highlighted. To evaluate the rapid protocol and the specific MBT-Sepsityper module also on rare yeast species and because of the small incidence of fungemia in our hospital, we used spiked blood culture. Therefore, the number of strains tested for each species did not reflect the real fungal epidemiology of our hospital as we wanted Consequently, according to our results and to our current epidemiology of fungemia, an increase of reliable identification rates is expected in clinical situation with the rapid protocol.

One of the main advantages of rapid MALDI-TOF-MS protocols for the diagnosis of BSI is the potential identification of more than 2200 different species present in the Bruker database. Indeed, rare, uncommon and fastidious or slow growing bacteria like *Finegoldia magna* have been detected in our study that wouldn’t have been identified with other molecular techniques or would have require 2 or 3 days of incubation on agar subcultures. RS protocol misidentified only 3/299 samples. In two cases, the erroneous identification (*Lactobacillus paralimentarius* instead of *Staphylococcus epidermidis* and *Aeromonas veronii* instead of *Staphylococcus haemolyticus*) could have been rejected based on prior Gram stain result of the positive blood culture and may have been consecutive to insufficient cleaning of MALDI TOF re-usable target. For the last case, a different species of coagulase-negative *Staphylococcus* was identified (*S. hominis* instead of *S. pasteuri*) which wouldn’t have led to an erroneous treatment decision as recommendations are the same for the management of all coagulase-negative *Staphylococcus* in positive blood cultures [74, 75]. Among unidentified bacteria, we assume the high proportion of unidentified *Enterobacterales* to be randomly related to red blood cells lysis or centrifugation issues observed with some samples, while we hypothesize a lower organism load or biomass in the positive blood cultures as the main cause for unidentification of anaerobes and GP cocci, as it has already been shown [76]. Importantly, on the 13 polymicrobial blood culture (with at least 2 different bacteria), neither RS ± FA nor SS protocols allowed a complete identification, while it may be possible with multiplex PCR [36]. For 9 of them (69%), RS ± FA identified one bacterium present in the bottle. Higher identification rate for polymicrobial samples using Sepsityper® kit have been observed in other studies (34.3% to 83.8%), but these studies considered to have a reliable identification if one bacteria was reliably identified [45, 57, 77]. These results emphasize the importance of still performing a Gram stain prior to the RS ± FA protocol.

Further reduction of technical time of this rapid protocol seems possible by processing several positive samples in small series and by performing identification only on the first positive blood culture for a bacteremic episode. Automatization (not yet available) of the RS ± FA or SS process would allow running all the samples as soon as they are positive. Other limitations of this approach exist. Despite a quick and reliable identification, and a low cost (around 10€/positive blood culture), RS ± FA protocol do not allow quick antimicrobial susceptibility testing nor resistance genes detection. However, several screening assays have been designed to identify in 15 min to 1 h the hydrolysis of third generation cephalosporins or carbapenemes in GN bacteria and can be performed on the pellet obtained at the end of the RS ± FA protocol [78–80].

## Conclusion

In conclusion, Rapid Sepsityper® protocol is an interesting commercial assay for quick bacterial identification in BSI that could allow early adaptation of empirical antibiotic treatment according to the species identified. RS ± FA protocol associated to MBT-Sepsityper module provide the fastest results among available commercial assays with reliable bacterial identification to the species level in more than 2/3 of the samples (68.6%) in 10 min. When using MALDI-TOF cut-offs defined by the manufacturers, the sensibility of Rapid Sepsityper® protocol remains however on average 10–15% lower than several in-house protocols but this assay does not require on site validation. Its low turnaround time will may help laboratories to implement this assay in their routine diagnosis protocols. In case of failure, it can easily be continued with the SS protocol which allowed 10% additional identification while being only done on 1/3 of the samples, thus reducing global hands on time of the technique. Specific MBT-Sepsityper module increases reliable identification rate of rapid Sepsityper® protocol and may also provide higher identification rates if evaluated with other in-house protocols compared to currently published results. As regards yeast identification, RS or RS + FA remain less effective than SS protocol. MBT-Sepsityper module increased yeast identification rate to current standard but with an identification obtained in 10 min using the RS + FA protocol, and increased identification rate up to 90% with the SS protocol.

## Supplementary Information

**Additional file 1: Figure S1.** Percentage of reliable bacteria identification according to the type of blood culture bottle using the rapid diagnosis strategy (Rapid Sepsityper® with or without formic acid protocols: RS ± FA). (N: number of samples tested; ns: no statistically significant difference; *: p < 0.05)

## Data Availability

The datasets used and/or analyzed during the current study are available from the corresponding author on reasonable request.

## Abbreviations

ACN: Acetonitrile
BSI: Bloodstream infection
CoNS: Coagulase negative Staphylococci
GN: Gram negative
GP: Gram positive
FA: Formic acid
MALDI-TOF MS: Matrix Assisted Laser Desorption Ionization-Time of Flight mass spectrometry
MDR: Multidrug resistant
MRSA: Methicillin resistant *Staphylococcus aureus*
N: Number
PCR: Polymerase Chain Reaction
PM: Polymicrobial
PNA-FISH: Peptide Nucleic Acid Fluorescence In Situ Hybridization
RS: Rapid Sepsityper®
SDS: Sodium dodecyl sulfate
SS: Standard Sepsityper®
TFA: Trifluoroacetic acid
